# Lack of Correlation Between Intracranial Carotid Artery Modified Woodcock Calcification Score and Prognosis of Patients With Acute Ischemic Stroke After Intravenous Thrombolysis

**DOI:** 10.3389/fneur.2019.00696

**Published:** 2019-07-02

**Authors:** Xin-Wei He, Rong Zhao, Ge-Fei Li, Bo Zheng, Yi-Lan Wu, Yan-Hui Shi, Yi-Sheng Liu, Mei-Ting Zhuang, Jia-Wen Yin, Guo-Hong Cui, Jian-Ren Liu

**Affiliations:** ^1^Department of Neurology, Shanghai Ninth People's Hospital, Shanghai Jiao Tong University School of Medicine, Shanghai, China; ^2^Clinical Research Center, Shanghai Jiao Tong University School of Medicine, Shanghai, China

**Keywords:** acute ischemic stroke, intracranial carotid artery, calcification, modified Woodcock calcification score, prognosis

## Abstract

There have been few studies about the association between intracranial carotid artery calcification (ICAC) and acute ischemic stroke (AIS) prognosis after intravenous thrombolysis (IVT). We aimed to analyze the association between ICAC and prognosis (including symptomatic intracranial hemorrhage (sICH), functional outcome and death) of AIS patients treated with IVT. In this retrospective study, we consecutively included 232 AIS patients treated with IVT between April 2012 and December 2018. ICAC was evaluated using the modified Woodcock calcification visual score on non-enhanced cranial computed tomography scans. Poor functional outcome was defined as a modified Rankin Scale score > 2 at 3 months. We found that the modified Woodcock calcification score was associated with ICH, poor outcome, and death in univariable analyses on the symptomatic side and/or bilaterally. However, after adjustment for other different covariates, the results showed no significant difference. We documented that the presence and severity of ICAC did not significantly modify the beneficial effects of rtPA treatment in AIS.

## Introduction

Early recanalization therapy is recognized as the only effective method to decrease disability and mortality due to acute ischemic stroke (AIS), and it mainly includes arterial intervention and intravenous thrombolysis (IVT) (1, 2). IVT using recombinant tissue-type plasminogen activator (rtPA) is still considered the first recommended treatment because of its convenience, minimal trauma, rapid action, and low cost (2–4).

Artery calcification is one of the constituents of atherosclerotic plaques and has been used as a marker of the presence, severity and prognosis of atherosclerosis (5, 6). For example, the presence of coronary artery calcification is a well-known potent predictor of the prognosis of coronary heart disease (CHD) and other vascular events (5, 7).

The development of neuroimaging technology has provided an objective diagnosis and treatment basis for many nervous system diseases (8, 9), but there are fewer data from neuroimaging studies about the clinical prognosis of AIS patients treated with IVT. Multiple studies have demonstrated that the presence of intracranial carotid artery calcification (ICAC) is an independent risk factor for cerebral ischemic symptoms, and it is also an independent predictor of stroke recurrence and death (10, 11). Until now, the impact of ICAC on the prognosis of AIS patients treated with IVT has been uncertain. Cranial computed tomography (CT) scans are routinely used before rtPA treatment in AIS patients and can reliably detect ICAC (2, 12). If ICAC detection on cranial CT is related to prognosis, it could have great clinical importance for promoting decision making in the treatment of AIS patients.

The presence of calcification of the carotid siphon is defined as the portion of internal carotid artery stenosis between the petrous apex and anterior clinoid, and it is an indicator of internal carotid artery stenosis (13, 14). The visual modified Woodcock carotid artery calcification score has very good correlations with the semiautomated quantitative Agatston score and calcium volume evaluation (15, 16), which are both considered quick and reliable for quantifying ICAC.

Therefore, the present study aimed to analyze the association between the modified Woodcock calcification score and prognosis (including symptomatic intracranial hemorrhage (sICH), functional outcome and death) of AIS patients treated with IVT.

## Materials and Methods

### Study Patients

This was a retrospective study of AIS patients who were treated with IVT between April 2012 and December 2018 at the Department of Neurology of the Ninth People's Hospital Affiliated to Shanghai Jiao Tong University School of Medicine. The indication and contraindication for IVT were conducted according to the Guidelines for the early management of patients with acute ischemic stroke from the American Heart Association/American Stroke Association (1, 2, 4, 12).

**Figure 1:**
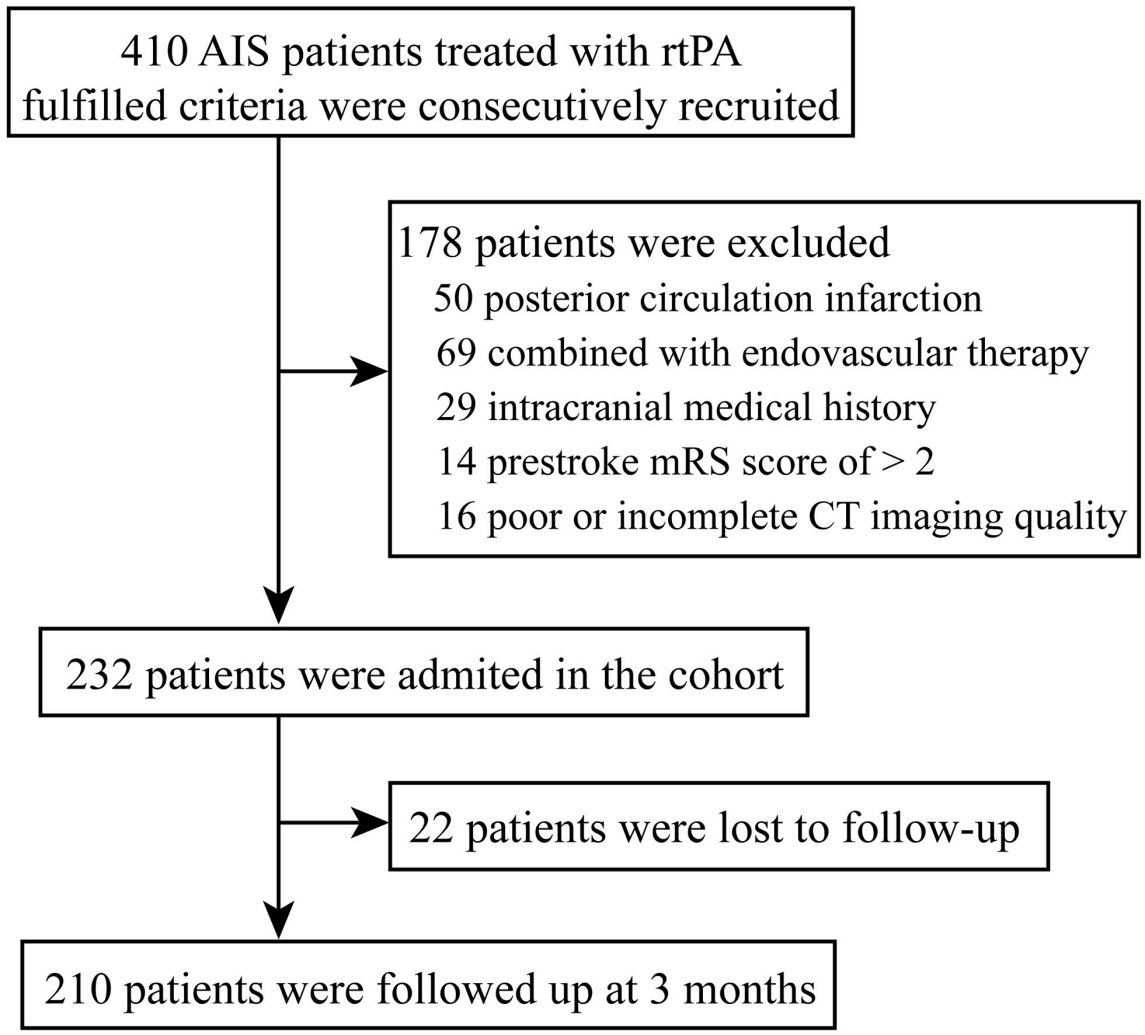
Study flow chart. AIS, acute ischemic stroke; rt-PA, recombinant tissue-type plasminogen activator; mRS, modified Rankin scale; CT, computed tomography.

During the study period, a total of 410 patients were submitted to IVT in our center. In the present study, the reasons for exclusion were: (1) posterior circulation infarction (*n* = 50), (2) combined with endovascular therapy (*n* = 69), (3) intracranial medical history (including history of ischemic stroke, hemorrhagic stroke, or space-occupying lesions) (*n* = 29), (4) prestroke mRS score of > 2 (*n* = 14), (5) poor or incomplete CT imaging quality (*n* = 16) (Figure 1).

This study was approved by the ethic committee of Shanghai Ninth People's Hospital, Shanghai Jiao Tong University School of Medicine. The study protocol conforms to the ethical guidelines of the 1975 Declaration of Helsinki. Informed consent was exempted by the committee because of the retrospective study based on routine clinical data (Ethical approval number: 2016-221-T170).

### Clinical Protocol

IVT was done with rtPA (Actilyse, Boehringer Ingelheim, Germany), according to internationally recognized guidelines (1, 2, 4, 12), at a dose of 0.9 mg/kg. Ten percent of the total dose were given intravenously as bolus over 1 min and the remaining 90% were given as infusion using an electronic perfusion device over 1 h. The patients were monitored during the procedure for any adverse reaction and complication. Vitals were monitored every 15 min during the procedure, then every hour for 2 h, and finally every 2 h for the following 24 h. All patients underwent a cranial CT scan at 24 h after thrombolysis or whenever neurological worsening occurred to evaluate the presence of intracranial hemorrhage.

Additionally, T1 and T2 weighted magnetic resonance (MR) imaging, diffusion-weighted MR imaging, and/or fluid-attenuated inversion recovery (FLAIR), and at least one kind of cerebral angiography (MR angiography, CT angiography or digital subtraction angiography), carotid ultrasound examination, and electrocardiogram were performed in the following days.

### Data Collection and Determination

Age, gender, medical history of hypertension, diabetes, CHD and atrial fibrillation, smoking and drinking, associated laboratory tests and imaging information were retrieved from our medical institutional database. Risk factors were defined as follows: hypertension (systolic blood pressure / diastolic blood pressure ≥ 140/90 mmHg, a history of hypertension or by the use of antihypertensive treatment), diabetes mellitus (fasting blood glucose level ≥ 7.0 mmol/L, a history of diabetes, or by the use of diabetic medications), dyslipidemia (serum triglycerides > 1.7 mmol/L, low-density lipoprotein > 3.4 mmol/L, high-density lipoprotein cholesterol < 0.8 mmol/L, or by the use of lipid-lowering agents), smoking (smoked at the time of stroke or had quit smoking within 1 year of the stroke), and drinking (>2 standard alcoholic beverages consumed per day).

Stroke was classified according to the Trial of ORG 10172 in Acute Stroke Treatment (TOAST) classification: (1) large artery atherosclerosis (LAA); (2) small vessel occlusion (SVO); (3) cardioembolism (CE); (4) stroke of other determined etiology; and (5) stroke of undetermined etiology (17). The National Institute of Health Stroke Score (NIHSS) was determined as previously reported (18). Moderate-to-severe stroke was defined as an NIHSS score ≥8 on admission, consistent with the existing literature (19, 20) sICH was defined according to the European Cooperative Acute Stroke Study (ECASS) III criteria (21).

Clinical functional outcome was assessed at 3 months after stroke by a specialized research nurse who was blinded to the carotid artery calcification scores. The modified Rankin Scale (mRS) score was evaluated. mRS ≤ 2 indicated a good outcome, while mRS > 2 indicated a poor outcome (14).

### Modified Woodcock Calcification Scoring

All included patients had available non-contrast cranial CT axial scan (Philips Brilliance 64 CT Scanner; Philips Healthcare, Andover, MA, USA) with slice thickness of 5 mm at admission (routine practice in China).

**Figure 2:**
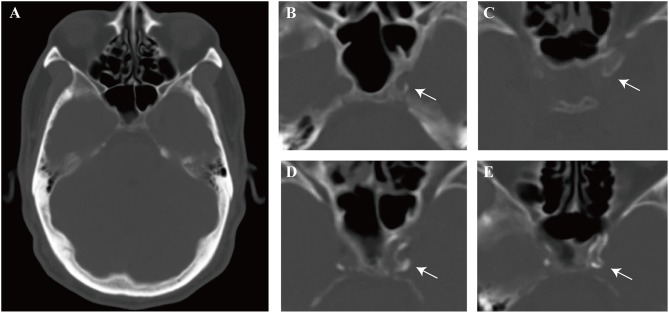
Schematic representation of calcification patterns on bone window of computed tomography (CT). **(A)** One of the interested axial slices on carotid siphon. **(B–E)** Modified Woodcock visual calcium score. **(B)** Thin, discontinuous calcification, score = 1, **(C)** Thin, continuous calcification, score = 2, **(D)** Thick, discontinuous calcification, score = 2, **(E)** Thick, continuous calcification, score = 3. Arrow indicates calcification. The scores assigned for each axial slice were finally totaled to create a total score.

We detected ICAC scores according to the modified version of the Woodcock visual scoring (15, 16). In brief, the ICAC score of carotid siphon on each axial CT slice was defined as follows, 0 for no ICAC; 1 for thin, discontinuous ICAC; 2 for thin, continuous ICAC or thick, discontinuous ICAC; 3 for thick, continuous ICAC (Figure 2). The score assigned for each axial slice was finally added up to create a total score for each ICA. Carotid siphon was defined as the portion between the petrous apex and anterior clinoid (13). Two independently neurologists blinded to the clinical data of the patients evaluated the bilateral calcification score of each slice in the Picture Archiving and Communication System (PACS) workstation, using a fixed bone window setting (window level of 500 HU and window width of 2000HU). They had received standardized training before CT images evaluation. The intraclass correlation coefficient (ICC) values of right and left side scores from the two observers were 0.851 (*P* < 0.001) and 0.956 (*P* < 0.001).

### Statistical Analyses

Continuous data with a normal distribution were presented as mean ± standard deviation (SD). Continuous without a normal distribution were presented as median (interquartile range). Categorical data were presented as frequency and percentage. The differences of scores between the two observers were analyzed by the ICC method. For univariate analyses, unpaired Student's *t*-test, Mann–Whitney *U* test or Pearson's chi-square test were used as appropriate. Sensitivity analysis was performed to deal with the patients lost to follow-up. Multivariate analyses were performed using logistic regression models, adjusted for potential influencing factors selected based on univariate analyses. The results are expressed as adjusted odds ratios (AORs) along with the corresponding 95% confidence intervals (95% CIs). All data were analyzed using SPSS 22.0 (IBM, Chicago, IL, USA). Two-sided *P* < 0.05 were considered statistically significant.

## Results

### Characteristics of the Patients

Eventually, 232 patients with AIS were included in this study (Figure 1). Basic characteristics of the patients are shown in Table 1. The age was 68.0 (59.3, 79.8) years old, and 59.1% of the patients were male. Considering the TOAST criteria, 33.6% of the patients had LAA stroke, 21.6% had CE stroke, 27.2% had SVO stroke, and 17.7% had other determined/undetermined etiologies.

**Table 1:** Baseline characteristics of patients.

	**All patients** **(*n* = 232)**	**mRS ≤ 2** **(*n* = 128)**	**mRS > 2** **(*n* = 82)**	***P*-value**
**Demographic data**				
Age, years	68.0 (59.3, 79.8)	63.0 (58.0, 72.0)	79.0 (70.0, 84.0)	< 0.001
Male, *n* (%)	137 (59.1)	90 (70.3)	31 (37.8)	< 0.001
**Stroke risk factors**, ***n*** **(%)**				
Hypertension	211 (90.9)	114 (89.1)	76 (92.7)	0.383
Diabetes mellitus	96 (41.4)	40 (31.2)	44 (53.7)	0.001
Dyslipidaemia	134 (57.8)	80 (62.5)	40 (48.8)	0.069
Coronary heart disease	60 (25.9)	28 (21.9)	26 (31.7)	0.112
Atrial fibrillation	67 (28.9)	22 (17.2)	38 (46.3)	< 0.001
Smoking	73 (31.5)	54 (42.2)	13 (15.9)	< 0.001
Drinking	48 (29.7)	38 (29.7)	8 (9.8)	0.001
**Laboratory values**				
HbA1c, %	5.9 (5.5, 6.6)	5.8 (5.5, 6.4)	6.1 (5.6, 6.6)	0.068
Homocysteine, μmol/L	12.9 (9.8, 16.8)	12.5 (9.2, 16.6)	14.2 (10.0, 18.6)	0.277
Creatinine, μmol/L	85 (69, 100)	84 (70, 100)	93 (72, 111)	0.982
**Stroke evaluation**				
NIHSS before IVT, points	5.5 (3, 13)	4 (2, 8)	12 (5, 17)	< 0.001
ICH, *n* (%)	26 (11.2)	9 (7.0)	17 (20.7)	0.003
Time to rt-PA treatment, min	153 (107, 194)	159 (121, 200)	157 (107, 200)	0.724
**TOAST subtype**, ***n*** **(%)**				
Large-artery atherosclerosis	78 (33.6)	45 (35.2)	26 (31.7)	0.606
Cardioembolism	50 (21.6)	15 (11.7)	30 (36.6)	< 0.001
Small-vessel occlusion	63 (27.2)	49 (38.3)	7 (8.5)	< 0.001
Others	41 (17.7)	19 (14.8)	19 (23.2)	0.126

*Values are presented median (interquartile range) for continuous variables and number (percentages) for categorical variables*.

*The P values reflect comparisons between the two groups stratified by mRS*.

*mRS, modified Rankin Scale; HbA1c, glycated hemoglobin; NIHSS, National Institutes of Health Stroke Scale; IVT, intravenous thrombolysis; ICH, intracranial hemorrhage; rt-PA, recombinant tissue-type plasminogen activator; TOAST, Trial of Org 10172 in Acute Stroke Treatment*.

The total modified Woodcock score (sum of the bilateral sides) were 3.0 (1.1, 5.0), and the symptomatic side score was 1.5 (1.0, 2.5). In addition, the scores were higher in patients with LAA than in patients with other TOAST types [3.8 (2.0, 6.0) vs. 3.0 (1.0, 5.0), *P* = 0.017 for total scores; 2.0 (1.0, 3.0) vs. 1.5 (0.5, 2.0), *P* = 0.031 for symptomatic side scores].

### Modified Woodcock Score, Stroke Severity, and ICH

The NIHSS score was 5.5 (3, 13) before IVT. No correlation was found between modified Woodcock score and stroke severity as defined by NIHSS score (*P* = 0.319 for total scores; *P* = 0.779 for symptomatic side scores). In addition, no difference was found in modified Woodcock score between patients with a moderate-to-severe strokes and patients with a mild strokes (Table 2).

**Table 2:** Modified Woodcock scores stratified by clinical parameters.

**Characteristics**	**Total modified woodcock scores**			**Symptomatic side scores**		
	**Yes**	**No**	***P***	**Yes**	**No**	***P***
M-to-S stroke	3.5 (2.0, 5.5)	3.0 (1.0, 5.0)	0.201	2.0 (1.0, 3.0)	1.5 (1.0, 2.5)	0.505
ICH	4.5 (3.0, 6.0)	3.0 (1.0, 5.0)	0.029	2.0 (1.4, 3.0)	1.5 (1.0, 2.5)	0.094
sICH	4.0 (3.4, 5.9)	3.0 (1.0, 5.5)	0.234	2.3 (1.4, 2.6)	1.5 (1.0, 3.0)	0.196
Poor outcome	4.5 (3.0, 6.0)	3.0 (1.0, 5.0)	0.005	2.0 (1.4, 3.0)	1.5 (1.0, 2.5)	0.008
Mortality	5.0 (3.0, 6.0)	3.5 (1.8, 5.0)	0.060	2.5 (1.3, 3.0)	1.5 (1.0, 2.5)	0.022

*M-to-S, moderate-to-severe; NIHSS, National Institutes of Health Stroke Scale; ICH, intracranial hemorrhage; sICH, symptomatic intracranial hemorrhage*.

ICH complication was detected in 26 patients. Patients with ICH had higher total modified Woodcock scores than patients without ICH (*P* = 0.008, Table 2). However, after adjustment for other, different covariates (including atrial fibrillation and NIHSS scores before IVT; Supplementary Table 1), the result had no significant difference (*P* = 0.309, Table 3). Ten of the 26 patients with ICH had sICH. No difference was found in modified Woodcock scores between patients with and without sICH (Table 2).

**Table 3:** Adjusted association of Modified Woodcock scores to clinical parameters.

	**ICH**		**Poor outcome**		**Mortality**	
**Modified woodcock scores**	**OR (95% CI)**	***P***	**OR (95% CI)**	***P***	**OR (95% CI)**	***P***
Symptomatic side	/	/	1.15 (0.90, 1.46)	0.274	1.17 (0.97, 1.41)	0.099
Total	0.309 (0.94, 1.23)	0.309	1.08 (0.94, 1.24)	0.301	/	/

*Per unit increase of modified Woodcock scores*.

*Each parameter was adjusted for a differential set of potential confounders: ICH, adjusted for atrial fibrillation and NIHSS scores before IVT; Poor Outcome: adjusted for age, sex, diabetes mellitus, atrial fibrillation, smoking, drinking, and NIHSS scores before IVT; Mortality: adjusted for age, sex, dyslipidaemia, atrial fibrillation, smoking, and drinking, NIHSS before IVT*.

*NIHSS, National Institutes of Health Stroke Scale; ICH, intracranial hemorrhage; OR, odds ratio; CI, confidence interval; IVT, intravenous thrombolysis*.

### Modified Woodcock Score, Functional Outcome, and Mortality

A total of 210 patients (90.5%) were followed up at 3 months; 82 (39.0%) of them had poor outcomes. The basic characteristics of the two groups are shown in Table 1. Patients with poor outcomes had higher modified Woodcock scores than patients with good outcomes at 3 months (*P* = 0.005 for total bilateral scores and *P* = 0.008 for the scores from the symptomatic side; Table 2). Other different covariates in the univariate analysis were age, sex, NIHSS before IVT, diabetes mellitus, atrial fibrillation, smoking, and drinking (Table 1). Adjustment for the aforementioned covariates rendered the association between modified Woodcock score and outcome statistically non-significant (Table 3).

Three months after their strokes, 21 (10.0%) subjects had died. The modified Woodcock score on the symptomatic side was associated with mortality (*P* = 0.022, Table 2). However, after adjustment for other different covariates (including age, sex, NIHSS before IVT, dyslipidemia, atrial fibrillation, smoking, and drinking; Supplementary Table 2), the results showed no significant difference (Table 3). In a sensitivity analysis in which we assumed that the patients lost to follow-up had died, the results did not change substantially (*P* = 0.888 for total scores and *P* = 0.859 for symptomatic side scores).

## Discussion

In the present study, no associations were observed of the modified Woodcock carotid artery calcification score with stroke severity, sICH, functional outcome, and mortality in AIS patients with IVT after adjustment for potential confounders, either on symptomatic side or bilaterally. We documented that the presence of ICAC does not modify significantly the beneficial effects of rtPA treatment in AIS.

Some recent studies have identified factors associated with prognosis, such as age, severity of stroke, and subtype of stroke (22–24), which can provide some reference for the clinical prognosis of patients with AIS treated with IVT. Age has been shown to be one of the main factors leading to delays in decision making for thrombolysis therapy because of the comorbidities associated with age (25). Yaghi et al. (26) showed that patients < 80 years of age were more likely to respond to thrombolysis therapy after AIS. A number of previous studies have shown that the baseline NIHSS score is associated with the prognosis of AIS after IVT. Indeed, even patients with a poor prognosis (baseline NIHSS > 20) could benefit from IV rtPA therapy (22, 27). However, these factors are not specific to AIS patients treated with rtPA. Additional studies are still necessary to determine whether there are other, more relevant factors.

ICAC scores were higher in patients with LAA strokes, which understandably differs significantly from other etiological types of stroke. Previous studies have demonstrated that calcifications increase with atherosclerosis burden and vascular stenosis and aggravate cerebral ischemia symptoms (13, 14, 16, 28, 29). Therefore, for patients with large cerebral artery occlusions and high ICAC scores, we must consider the possibility of patients with LAA strokes, and acute occlusion could happen on the basis of intracranial artery stenosis, especially when the patient has no cardiac disease history. If there exists acute occlusion following intracranial stenosis, simple endovascular thrombectomy might not be sufficient, and further balloon angioplasty or stent implantation could be needed.

Although several studies have focused on the presence and load of ICAC in other recanalization therapies, including endovascular interventions, the results are still highly controversial. Lee et al. (30) reported that a high ICAC burden was associated with grave prognosis (mRS ≥ 5) after revascularization in patients with acute middle cerebral artery trunk occlusion. Hernandez-Perez et al. (31) showed that the presence of severe ICAC was associated with poorer functional outcomes after endovascular thrombectomy in patients with anterior circulation large artery occlusion. In contrast, another study concluded that extensive ICAC did not have an impact on reperfusion or clinical outcomes in AIS patients undergoing endovascular therapy (14). In addition, Compagne et al. (32) recently demonstrated that ICAC volume was not associated with functional outcomes after endovascular thrombectomy for AIS, but they found that medial or intimal calcification patterns influenced the effect (32). For the factors influencing endovascular intervention treatment to cover many aspects, including mechanical devices, operation technique, intervention time and periprocedural complications, it requires more comprehensive consideration.

Studies about the association between ICAC and the prognosis of AIS patients treated with IVT have been relatively few. Lin et al. (33) showed that AIS patients with moderate to severe ICAC were more at risk of ICH but not sICH than patients with no or minor ICAC. In addition, Tábuas-Pereira et al. (34) found that AIS patients with higher grades of ICAC have higher mortality rates following IVT. There are many discrepancies among other studies and ours, such as different ICAC assessment methods; e.g., both of the studies used the Total Carotid Siphon Calcification (TCSC) score (33, 34) but different inclusion and exclusion criteria. For example, Tábuas-Pereira et al. (34) included patients with posterior circulation infarctions. In addition, different grouping methods were used; e.g., Lin et al. (33) dichotomized patients into two groups according to TCSC scores rather than analyzing the scores as continuous variables.

The modified Woodcock scale method can be suitably applied in acute clinical settings because it can be assessed quickly and consistently (15, 16). In the present study, the modified Woodcock calcification score was associated with ICH, poor outcome, and death in univariable analyses but not in multivariable analyses. A number of factors could play a role in this lack of association; e.g., some variables include in the multivariable analysis model were potential covariates. These results further confirmed that ICAC might be considered a marker of systemic atherosclerosis and a condition that is closely related to stroke-related risk factors (35). Although the results should only be considered hypothesis-generating results rather than definitive results, and they require further research, our findings did not support that the presence and severity of ICAC affect rtPA treatment decisions for AIS patients.

ICAC leads to significant stenosis of the lumen, accompanied by more atherosclerotic changes and arterial tortuosity (13, 14, 36), which can affect the success of endovascular intervention. In this case, the difficulty of passing the siphon curve by the distal access catheter or the aspiration catheter is obviously increased. This difficulty must be considered before treatment, for example, to select material to improve support and to consider the combination of stent retriever and aspiration catheter. The previous literature has also documented that ICAC could be related to the poor prognosis of AIS patients after thrombectomy, especially the intimal calcification pattern, because calcification of the intima is more likely to lead to smaller lumen and larger local atherosclerotic plaque, and plaque rupture is more likely to occur during thrombectomy (32); therefore it is likely to increase the difficulty and complications of endovascular thrombectomy (37). ICAC does not affect thrombolytic drugs passing through the vascular lumen. Therefore, in patients with severe intracranial artery calcification and acute occlusion of the large intracranial artery, intravenous thrombolysis before endovascular intervention might be more necessary.

The present study has some limitations. Indeed, the number of patients eligible for evaluation was small, likely because of the limited time window for IVT after AIS onset and the inclusion and exclusion criteria of this study. A large-scale, multicenter investigation combined could be conducted to validate and refine the results of the present study, and subgroup analysis based on TOAST classification in such studies is a better method may lead to find some new findings. Second, different calcification distributions and calcification types are presumed to have different pathological mechanisms and thus could have distinct clinical consequences (32, 38). However, additional technical examinations (e.g., CT angiography) are required. Third, the vessel diameter in the carotid siphon is typically on the order of 4 to 5 mm; therefore, partial volume averaging might also represent a source of error (15). Thus, it should be considered when we used a thicker section thickness (5 mm). However, a thickness of 5 mm is suitable and extensively used in clinical practice because of its smaller radiation exposure and faster scanning speed. Future studies could examine the impact of imaging parameters on ICAC.

In conclusion, ICAC was not associated with the prognosis of AIS patients treated with IVT. The presence and severity of ICAC did not significantly modify the beneficial effects of rtPA treatment.

## Data Availability

The datasets generated for this study are available on request to the corresponding author.

## Ethics Statement

This study was approved by the ethic committee of Shanghai Ninth People's Hospital, Shanghai Jiao Tong University School of Medicine. The study protocol conforms to the ethical guidelines of the 1975 Declaration of Helsinki. Informed consent was exempted by the committee because of the retrospective study based on routine clinical data (Ethical approval number: 2016-221-T170).

## Author Contributions

X-WH, RZ, and G-FL carried out the studies, participated in collecting data, and drafted the manuscript. BZ, Y-LW, Y-HS, Y-SL, M-TZ, and J-WY performed the statistical analysis and participated in its design. G-HC and J-RL designed and supervised the study, and revised the manuscript. All authors read and approved the final manuscript.

### Conflict of Interest Statement

The authors declare that the research was conducted in the absence of any commercial or financial relationships that could be construed as a potential conflict of interest.
