# Supplementary material for: Lack of Correlation Between Intracranial Carotid Artery Modified Woodcock Calcification Score and Prognosis of Patients With Acute Ischemic Stroke After Intravenous Thrombolysis
Source: Front Neurol. 2019 Jul 2;10:696. doi: 10.3389/fneur.2019.00696 (PMC6614196; doi:10.3389/fneur.2019.00696)
Supplement: Supplementary file 1 [file Table_1.DOCX]

**Lack of correlation between intracranial carotid artery modified Woodcock calcification score and** **prognosis of patients with acute ischaemic stroke after intravenous thrombolysis**

**Running title**

Carotid calcification and prognosis of stroke

**Authors and Affiliations**

Xin-Wei He^† 1, 2^, Rong Zhao^†^ ^1, 2^, Ge-Fei Li^† 1, 2^, Bo Zheng ^1, 2^, Yi-Lan Wu ^1, 2^, Yan-Hui Shi ^1, 2^, Yi-Sheng Liu ^1, 2^, Mei-Ting Zhuang ^1, 2^, Jia-Wen Yin ^1, 2^, Guo-Hong Cui^* 1, 2^, Jian-Ren Liu^* 1, 2^

1. Department of Neurology, Shanghai Ninth People’s Hospital, Shanghai Jiao Tong University School of Medicine, Shanghai, PR China;

2. Clinical Research Center, Shanghai Jiao Tong University School of Medicine, Shanghai, PR China

^†^These authors contributed equally to this work.

^*^ Correspondence:

Dr. Jian-Ren Liu, [liujr021@sjtu.edu.cn](mailto:liujr021@sjtu.edu.cn); Dr. Guo-Hong Cui, [gh_cui@qq.com](mailto:gh_cui@qq.com).

**Supplementary Table 1. Comparison by** **ICH for patients**

|  | With ICH  (n = 206) | ICH  (n = 26) | *P* value |
| --- | --- | --- | --- |
| Demographic data |  | | |
| Age, years | 68.0 (58.0, 80.0) | 70.5 (61.5, 81.3) | 0.220 |
| Male, n (%) | 123 (59.7) | 14 (53.8) | 0.567 |
| Stroke risk factors, n (%) |  | | |
| Hypertension | 187 (90.8) | 24 (92.3) | 0.798 |
| Diabetes mellitus | 81 (39.3) | 15 (57.7) | 0.073 |
| Dyslipidaemia | 122 (59.2) | 12 (46.2) | 0.204 |
| Coronary heart disease | 50 (24.3) | 10 (38.5) | 0.119 |
| Atrial fibrillation | 55 (26.7) | 12 (46.2) | 0.039 |
| Smoking | 68 (33.0) | 5 (19.2) | 0.154 |
| Drinking | 44 (21.4) | 4 (15.4) | 0.479 |
| Laboratory values |  | | |
| HbA1c, % | 5.9 (5.5, 6.7) | 6.6 (5.5, 8.6) | 0.057 |
| Homocysteine, μmol/L | 12.7 (9.8, 16.7) | 12.7 (9.3, 19.5) | 0.758 |
| Creatinine, µmol/L | 84.0 (70.0, 100.0) | 90.0 (67.5, 109.5) | 0.360 |
| Stroke evaluation |  | | |
| NIHSS before IVT, points | 5 (3, 11) | 9 (5, 16) | 0.020 |
| Time to rt-PA treatment, min | 158 (110, 200) | 158 (112, 198) | 0.913 |

Values are presented median (interquartile range) for continuous variables and number (percentages) for categorical variables.

The *P* values reflect comparisons between the two groups stratified by ICH.

Abbreviations: mRS, modified Rankin Scale; HbA1c, glycated haemoglobin; NIHSS, National Institutes of Health Stroke Scale; ICH, intracranial haemorrhage; IVT, intravenous thrombolysis; rt-PA, recombinant tissue-type plasminogen activator.

**Supplementary Table 2. Comparison by mortality for patients**

|  | Survival  (n = 189) | Death  (n = 21) | *P* value |
| --- | --- | --- | --- |
| Demographic data |  | | |
| Age, years | 68.0 (58.5, 80.0) | 82.0 (71.0, 84.0) | < 0.001 |
| Male, n (%) | 114 (60.3) | 7 (33.3) | 0.018 |
| Stroke risk factors, n (%) |  | | |
| Hypertension | 171 (90.5) | 19 (90.5) | 1.000 |
| Diabetes mellitus | 73 (38.6) | 11 (52.4) | 0.222 |
| Dyslipidaemia | 113 (59.8) | 7 (33.3) | 0.020 |
| Coronary heart disease | 46 (24.3) | 8 (38.1) | 0.171 |
| Atrial fibrillation | 49 (25.9) | 11 (52.4) | 0.011 |
| Smoking | 66 (34.9) | 1 (4.8) | 0.003 |
| Drinking | 46 (24.3) | 0 (0) | 0.005 |
| Laboratory values |  | | |
| HbA1c, % | 6.0 (5.5, 6.9) | 5.9 (5.4, 6.6) | 0.868 |
| Homocysteine, μmol/L | 12.7 (9.5, 17.3) | 14.2 (11.5, 20.6) | 0.313 |
| Creatinine, µmol/L | 84.0 (70.0, 100.0) | 97.0 (73.3, 119.3) | 0.186 |
| Stroke evaluation |  | | |
| NIHSS before IVT, points | 5 (3, 11) | 16 (12, 20) | < 0.001 |
| Time to rt-PA treatment, min | 160 (110, 200) | 156 (120, 178) | 0.636 |

Values are presented median (interquartile range) for continuous variables and number (percentages) for categorical variables.

The *P* values reflect comparisons between the two groups stratified by mortality.

Abbreviations: mRS, modified Rankin Scale; HbA1c, glycated haemoglobin; NIHSS, National Institutes of Health Stroke Scale; ICH, intracranial haemorrhage; IVT, intravenous thrombolysis; rt-PA, recombinant tissue-type plasminogen activator.
